# Hormone replacement therapy before breast cancer diagnosis significantly reduces the overall death rate compared with never-use among 984 breast cancer patients

**DOI:** 10.1038/sj.bjc.6690543

**Published:** 1999-07

**Authors:** H Jernström, J Frenander, M Fernö, H Olsson

**Affiliations:** Department of Oncology, University Hospital of Lund, Lund, S-221 83, Sweden

**Keywords:** breast cancer, overall survival, HRT, oestrogen receptor, progesterone receptor

## Abstract

Nine hundred and eighty-four breast cancer patients were interviewed regarding exogenous hormonal use. This represents a random sample of breast cancer patients in Southern Sweden referred to the Department of Oncology at Lund for treatment between 1978 and 1997 (excluding 1980 and 1981) with a 100% follow-up. Ever-use of hormone replacement therapy (HRT) prior to diagnosis was significantly associated with a longer overall survival in women with their breast cancer diagnosed at ages 45 and above, relative risk (RR) of dying 0.73 (95% confidence interval (CI) 0.62–0.87; *P* = 0.0005). Ever use of HRT prior to breast cancer diagnosis was significantly positively associated with overall longer survival after adjustment for T-stage, N-stage, M-stage, year of diagnosis and age at diagnosis, RR of dying 0.78 (95% CI 0.65–0.93; *P* = 0.006). Hormone replacement therapy use and oestrogen receptor positivity were independently significantly associated with overall longer survival, *P* = 0.005 and *P* < 0.0001, respectively, in one model. HRT use and progesterone receptor positivity were also independently significantly associated with longer overall survival, *P* = 0.003 and *P* = 0.0003, respectively, in another model. The mode of diagnosis was known in 705 women. Mammography screening was not more common among HRT users compared with never-users, where this information was available. Both mammography screening and HRT use were independently associated with longer survival, *P* = 0.002 and *P* = 0.038 respectively. © 1999 Cancer Research Campaign


*British Journal of Cancer* (1999) **80**(9), 1453–1458
© 1999 Cancer Research Campaign
Article no. bjoc.1999.0543

### Hormone replacement therapy before breast cancer diagnosis significantly reduces the overall death rate compared with never-use among 984 breast cancer patients

**H Jernström, J Frenander, M Fernö and H Olsson**

Department of Oncology, University Hospital of Lund, S-221 83 Lund, Sweden

**Summary** Nine hundred and eighty-four breast cancer patients were interviewed regarding exogenous hormonal use. This represents a random sample of breast cancer patients in Southern Sweden referred to the Department of Oncology at Lund for treatment between 1978 and 1997 (excluding 1980 and 1981) with a 100% follow-up. Ever-use of hormone replacement therapy (HRT) prior to diagnosis was significantly associated with a longer overall survival in women with their breast cancer diagnosed at ages 45 and above, relative risk (RR) of dying 0.73 (95% confidence interval (CI) 0.62–0.87; $P = 0.0005$). Ever use of HRT prior to breast cancer diagnosis was significantly positively associated with overall longer survival after adjustment for T-stage, N-stage, M-stage, year of diagnosis and age at diagnosis, RR of dying 0.78 (95% CI 0.65–0.93; $P = 0.006$). Hormone replacement therapy use and oestrogen receptor positivity were independently significantly associated with overall longer survival, $P = 0.005$ and $P < 0.0001$, respectively, in one model. HRT use and progesterone receptor positivity were also independently significantly associated with longer overall survival, $P = 0.003$ and $P = 0.0003$, respectively, in another model. The mode of diagnosis was known in 705 women. Mammography screening was not more common among HRT users compared with never-users, where this information was available. Both mammography screening and HRT use were independently associated with longer survival, $P = 0.002$ and $P = 0.038$ respectively.

**Keywords**: breast cancer; overall survival; HRT; oestrogen receptor; progesterone receptor

Hormone replacement therapy (HRT) is used by an increasing number of women to relieve menopausal problems. A protective effect against bone loss and cardiovascular disease has been demonstrated. One study has reported that oestrogen replacement therapy prolonged survival in women when coronary artery disease was present, whereas less effect was seen in absence of coronary artery disease (Sullivan et al, 1990). However, in a randomized placebo controlled trial of HRT in women with coronary heart disease, there was no significant difference between groups in occurrence of non-fatal myocardial infarction or coronary heart disease (Hulley et al, 1998). Concern has been raised about an increased incidence of breast cancer after HRT use, especially after more than 10 years of use (Grodstein et al, 1997). In the collaborative re-analysis of 51 studies the breast cancer risk was found to be increased by 2.3% per year of use and to be greatest during and shortly after discontinuation of HRT (Collaborative Group on Hormonal Factors in Breast Cancer, 1997). In general, no increased mortality due to breast cancer in women who have used HRT has been shown (Hunt et al, 1990; Henderson et al, 1991; Yuen et al, 1993; Willis et al, 1996). However, concern has been raised after long-term (>10 years) use (Grodstein et al, 1997). Less advanced clinical stage at breast cancer diagnosis among HRT users has been reported by three studies and may account for the decreased mortality rate in some studies (Brinton et al, 1986; Bergkvist et al, 1989; Magnusson et al, 1996). The less advanced clinical stage may be due to surveillance bias (Schairer et al, 1994), a healthy oestrogen user bias (Yuen et al, 1993), selection bias (Henderson et al, 1991), or a modifying effect from HRT on the tumour biology (Squitieri et al, 1994; Bonnier et al, 1995; Salmon et al, 1995; Harding et al, 1996; Magnusson et al, 1996).

In observational studies, HRT has been shown to reduce the risk of cardiovascular disease; this was, however, not confirmed in a randomized placebo controlled trial (Hulley et al, 1998). Other positive effects such as decreased risk of osteoporosis, while negative effects such as an increased risk of breast cancer have also been demonstrated (Collaborative Group on Hormonal Factors in Breast Cancer, 1997). We have studied the total effect on survival in 984 women with breast cancer, comparing women who ever used HRT before diagnosis with never users in relation to overall survival. Tumour oestrogen receptor (ER) and progesterone receptor (PR) status, tumour stage and the mode of diagnosis have also been investigated and related to ever-use of HRT before diagnosis and overall survival.

### MATERIALS AND METHODS

Approximately 25–33% of all breast cancer patients from the south Swedish health care region are referred to the University Hospital of Lund, including all stages of breast cancer. Patients are referred to the hospital and randomly allocated to any of the oncologists working with breast cancer. This material therefore consists

*Received 7 July 1998*
*Revised 13 January 1999*
*Accepted 29 January 1999*

*Correspondence to:* H Olsson

**Table 1** Overall survival studied among a random consecutive sample of breast cancer patients from southern Sweden, diagnosed between 1978 and 1997

| | All ($n$ = 984) | | HRT users ($n$ = 135) | | Never-users ($n$ = 849) | |
|---|---|---|---|---|---|---|
| | **Mean** | **(Range)** | **Mean** | **(range)** | **Mean** | **(Range)** |
| Age at diagnosis | 59.9 | (45–87) | 60.3 | (45–83) | 59.8 | (45–87) |
| Days of survival | 2875 | (0–6687) | 2953 | (86–6415) | 2862 | (0–6687) |
| Age at menopause | 49.7 | (30–62) | 49.5 | (30–57) | 49.7 | (30–62) |
| HRT (months) | | | 60.3 | (1–240) | 0 | |
| | *n* | (%) | *n* | (%) | *n* | (%) |
| Detected by | | | | | | |
| Screening | 121 | (12.3) | 24 | (17.8) | 97 | (11.4) |
| Symptoms | 590 | (60.0) | 100 | (74.1) | 490 | (57.7) |
| Missing | 273 | (27.7) | 11 | (8.1) | 262 | (30.9) |
| Oestrogen receptor | | | | | | |
| Positive | 486 | (49.4) | 70 | (51.9) | 416 | (59.0) |
| Negative | 244 | (24.8) | 27 | (20.0) | 217 | (25.6) |
| Missing | 254 | (25.8) | 38 | (28.1) | 216 | (25.4) |
| Progesterone receptor | | | | | | |
| Positive | 360 | (36.6) | 54 | (40.0) | 306 | (36.0) |
| Negative | 299 | (30.4) | 36 | (26.7) | 263 | (31.0) |
| Missing | 325 | (33.0) | 45 | (33.3) | 280 | (33.0) |

Characteristics of the women included in this study are shown. There were no significant differences between HRT users and never-users when comparing mode of diagnosis ($P$ = 0.44), oestrogen receptor status ($P$ = 0.21) and progesterone receptor status ($P$ = 0.27).

of a randomly selected consecutive number of patients from the region between 1978 and 1997 (excluding 1980 and 1981). Relatively fewer very old patients are referred for radiation treatment. Lund is the only hospital giving radiation treatment in the region (excluding Malmö city). The main reason for not referring patients for radiation treatment is due to randomized trials, where treatment arms are excluding radiation treatment in stage 1 and stage 2 patients. The present study is, therefore, based on a random sample of approximately 11% of all breast cancer patients from the south Swedish health care region during this period.

From November 1978 to spring of 1997 (excluding 1980 and 1981) all breast cancer patients seen by one of us (HO) were interviewed. This study has been approved by the Ethical Committee for Medical Research at the University of Lund. Information obtained included age, previous cancers, menopausal status, age at menopause and use of HRT, as well as physical characteristics such as height and weight. Information regarding date at diagnosis, and T-, N- and M-stage was obtained from the South Swedish Tumour Registry, clinical information and the cancer care programme. T- and N-staging was based on pathological post-surgical information. Women with previous breast cancers were excluded from this study as a previous breast cancer may have influenced the use of oestrogen compounds. Until spring 1997, a total of 984 breast cancer patients who were 45 years or older at diagnosis and without previous breast cancers had been interviewed. Eight hundred and forty-nine patients had never used HRT and 135 patients had ever used HRT prior to their breast cancer diagnosis. One hundred and thirteen women had ever used oestrogen replacement therapy, while 30 women had ever used a combined oestrogen–progestagen replacement therapy. Some women had used both oestrogen alone and a combination of oestrogen and progesterone. As there were too few women on the combined regimen the groups have been combined into an HRT group. The breast cancers were more often detected due to symptoms than screening. See Table 1 for characteristics of the women included.

In order to obtain time of survival from diagnosis, the 984 breast cancer patients were matched against the population census registry 1 March 1997, from which date of death was obtained. The follow-up was 100% complete.

ER and PR contents were measured with two different techniques: ER content was measured with isoelectric focusing in polysaccharide gels and enzyme immuno-assay, and PR content with enzyme immuno-assay and a dextran-coated charcoal method with Scatchard analysis (Fernö et al, 1989, 1983, 1986) enzyme immuno-assay was performed according to kit instructions (Abbot Laboratories, Diagnostic Division, Chicago, IL, USA). Samples with receptor content values of ≥ 10 (isoelectric focusing and dextran-coated charcoal method) or ≥ 25 (enzyme immuno-assay) fmol $mg^{-1}$ protein were classified as ER- or PR-positive, and samples with values below these levels as ER- or PR-negative (Sigurdsson et al, 1990). As different methods have been used for analyses of receptor content, an adjustment factor was applied, and ER and PR contents ≥ 25 were considered positive.

### Statistics

For univariate analyses of survival time in relation to HRT use, the Kaplan–Meier method and the log-rank test were used. For univariate analysis on receptor status and mode of diagnosis in relation to HRT the $\chi^2$ test was used. For the multivariate models also taking T-, N-, M-stage and age of diagnosis into account, a Cox regression model was used. The assumption of a proportional hazard was approximately valid. These calculations were done by the SPSS statistical program. Test for linear trend between HRT-use and T-stage was also done.

 © 1999 Cancer Research Campaign

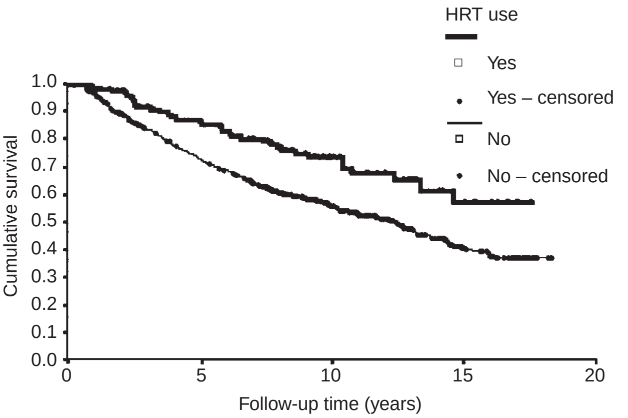

**Figure 1** Overall survival time in relation to hormone replacement therapy (HRT) use in breast cancer patients diagnosed at age 45 years or older. Women who had ever used HRT before diagnosis had a significantly higher overall survival (log-rank $P = 0.0004$, Kaplan–Meier)

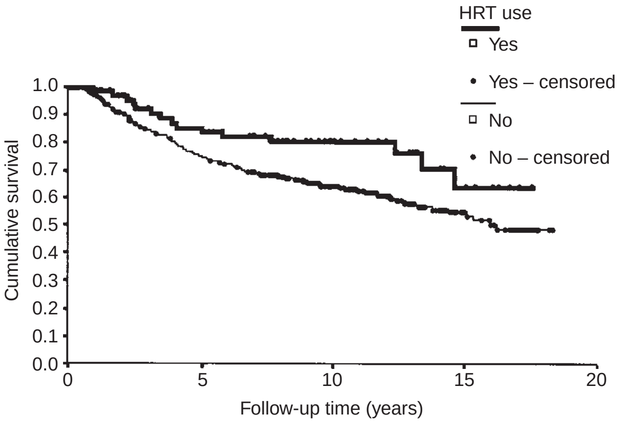

**Figure 2** Overall survival time in relation to hormone replacement therapy (HRT) use in breast cancer patients diagnosed between 45 and 60 years of age. Women who had ever used HRT before diagnosis had a significantly higher overall survival (log-rank $P = 0.018$, Kaplan–Meier)

**Table 2** Overall survival studied among a random consecutive sample of breast cancer patients from southern Sweden, diagnosed between 1978 and 1997 and treated at Lund University Hospital

| Variable | B | s.e.m. | Wald | *P*-value | R | OR | (95% CI) |
|---|---|---|---|---|---|---|---|
| HRT | –0.25 | 0.09 | 7.47 | 0.006 | –0.03 | 0.78 | (0.65–0.93) |
| T-stage (high/low) | 0.30 | 0.06 | 27.31 | 0.000 | 0.07 | 1.34 | (1.20–1.50) |
| N-stage (high/low) | 0.37 | 0.06 | 38.36 | 0.000 | 0.07 | 1.45 | (1.29–1.62) |
| M-stage (high/low) | 0.51 | 0.11 | 23.92 | 0.000 | 0.06 | 1.66 | (1.34–2.06) |

Cox regression model including 949 of the 984 women with breast cancer diagnosis at age 45 years or older, analysing the relationship between HRT use and time of survival after breast cancer diagnosis; 410 women had died. HRT use was significantly associated with lower risk of dying ($P = 0.006$) after adjustment for age and year of diagnosis.

**Table 3** Overall survival studied among a random consecutive sample of breast cancer patients from Southern Sweden, diagnosed between 1978 and 1997 and treated at Lund University Hospital

| Variable | B | s.e.m. | Wald | *P*-value | R | OR | (95% CI) |
|---|---|---|---|---|---|---|---|
| HRT | –0.26 | 0.14 | 3.49 | 0.061 | –0.03 | 0.77 | (0.59–1.01) |
| T-stage (high/low) | 0.25 | 0.08 | 8.91 | 0.000 | 0.06 | 1.28 | (1.09–1.51) |
| N-stage (high/low) | 0.57 | 0.09 | 37.49 | 0.000 | 0.13 | 1.77 | (1.47–2.12) |
| M-stage (high/low) | 0.77 | 0.18 | 19.02 | 0.000 | 0.09 | 2.16 | (1.53–3.05) |

Cox regression model including 520 of the 538 women with breast cancer diagnosis at age 45–60 years, analysing the relationship between HRT use and time of survival after breast cancer diagnosis. 184 women had died. HRT use was associated with lower risk of dying ($P$=0.061), after adjustment for age and year of diagnosis.

### RESULTS

Four hundred and thirty out of the 984 patients included in this study had died. Among the 849 non-users, 395 women had died (46.5%), and among the 135 HRT-users 35 had died (25.9 %). No significant correlations were seen between mode of diagnosis and HRT use, or height and weight and HRT-use.

In a univariate model including all 984 women, 135 ever HRT users and 849 never-users, HRT use was significantly positively associated with a longer survival time ($P = 0.0004$) (Figure 1). After adjustment for year and age of diagnosis, the relative risk (RR) of dying for HRT users was 0.74 (95% confidence interval (CI) 0.62–0.87; $P = 0.0005$). A Cox regression model ($n = 949$) also taking T-, N-, M-stage, year and age at diagnosis into account, showed that HRT users had a significantly improved overall survival after diagnosis, RR of dying 0.78 (95% CI 0.65–0.93; $P = 0.006$). T-, N-, M-stage and age at diagnosis were all significantly associated with time of survival ($P < 0.0001$) respectively. In order to be able to calculate 95% CIs we divided T-stage into low and high T-stage, defining T-stage 1 as low and T-stages 2–4 as high. For N-stage, we defined N-stage 0 as low and N-stages 1–3 as high (Table 2). When including height and weight into the model, these two factors were not significantly associated with overall survival and did not essentially alter the results.

For 5 or more years of HRT use ($n = 892$) the RR of dying was 0.77 (95% CI 0.56–1.08) and for fewer than 5 years of HRT use ($n = 935$) RR 0.78 (95% CI 0.63–0.96) compared with never-users, after adjustment for high T-, N-, M-stage and age at diagnosis. Duration of HRT use was known in 129 of the 135 women who were ever-users.

As breast cancer would be the main cause of death in women between 45 and 60 years of age, we narrowed the age-span to women with age of diagnosis between these ages. In a univariate model including 538 women, of whom 74 were ever-users and 464 never-users, HRT use was significantly positively associated with a longer survival time ($P = 0.0177$) (Figure 2). Using the regression model also taking T-, N-, M-stage, year of diagnosis and age at diagnosis into account ($n = 520$), showed that HRT users had a trend toward improved overall survival after diagnosis RR of dying 0.77 (95% CI 0.59–1.01; $P = 0.0611$). T-stage ($P = 0.0028$), N-stage ($P < 0.0001$) and M-stage ($P < 0.0001$) were significantly associated with overall survival, but not with either year of diagnosis ($P = 0.90$) or age at diagnosis ($P = 0.34$). In order to be able to calculate 95% CIs we divided T-, N-, M-stage into low and high

*© 1999 Cancer Research Campaign*

**Table 4** Comparing T- and N-stage in women with their breast cancer detected at age 45 years or above in a random consecutive sample of breast cancer patients from southern Sweden, diagnosed between 1978 and 1997 and treated at Lund University Hospital

| | Never-users | HRT users | Percent HRT users |
|---|---|---|---|
| T-stage | | | |
| 1 | 357 | 73 | 17.0 |
| 2 | 355 | 48 | 11.9 |
| 3 | 72 | 8 | 10.0 |
| 4 | 54 | 5 | 8.5 |
| N-stage | | | |
| Negative | 318 | 55 | 14.7 |
| Positive | 512 | 77 | 13.1 |

Women who ever used HRT were more likely to have their tumour detected at an earlier T-stage, test for linear trend (*P* = 0.0068), whereas no difference was found between N-stages. N-stages 1–3 were analysed as lymph node positive as too few women had stage 2 and 3 for meaningful comparisons.

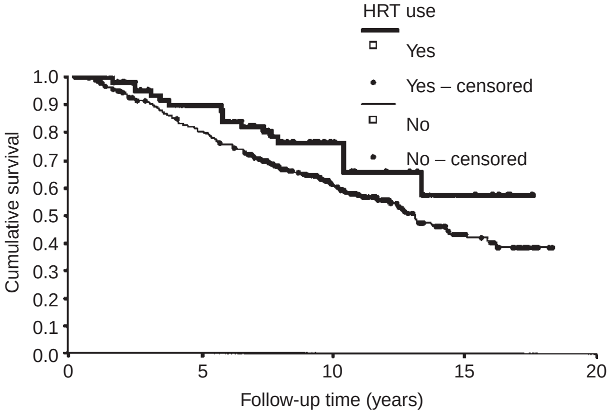

**Figure 3** Overall survival time in relation to hormone replacement therapy (HRT) use in oestrogen receptor-positive breast cancer patients, aged 45 years and older. HRT use was borderline significantly associated with longer survival (log-rank *P* = 0.050, Kaplan–Meier)

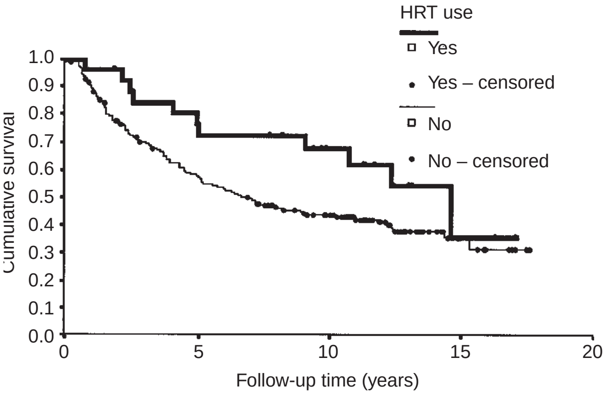

**Figure 4** Overall survival time in relation to hormone replacement therapy (HRT) use in oestrogen receptor-negative breast cancer patients, aged 45 years and older. HRT use was non-significantly associated with longer survival (log-rank *P* = 0.061, Kaplan–Meier)

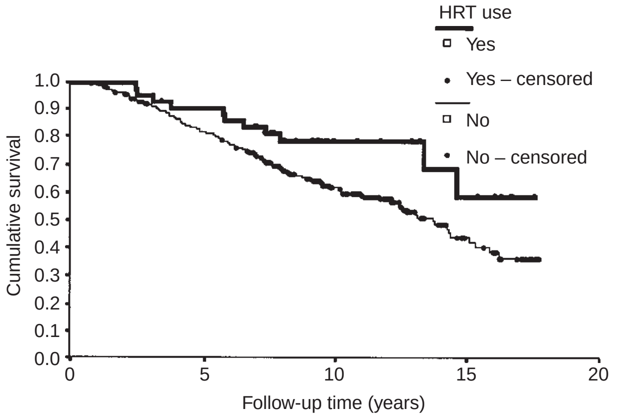

**Figure 5** Overall survival time in relation to hormone replacement therapy (HRT) use in progesterone receptor-positive breast cancer patients, aged 45 years and older. HRT use was significantly associated with longer survival (log-rank *P* = 0.037, Kaplan–Meier)

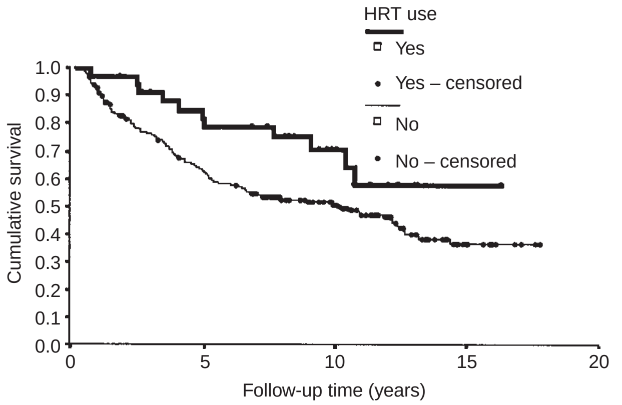

**Figure 6** Overall survival time in relation to hormone replacement therapy (HRT) use in progesterone receptor-negative breast cancer patients, aged 45 years and older. HRT use was significantly associated with longer survival (log-rank *P* = 0.035, Kaplan–Meier)

as described above (Table 3). When entering height and weight into the model, a short height was significantly associated with shorter overall survival ($P$ = 0.02) and so was a heavy weight ($P$ = 0.002), while the results from the other factors remained essentially the same. There was no significant difference in time of survival depending on whether a woman had used HRT for 5 years or more, versus fewer than 5 years after adjustment for high T-, N-, M-stage and age at diagnosis.

Comparing the influence of HRT on T-stage and N-stage among women with breast cancer diagnosed at age 45 years or older showed that HRT was associated with a lower T-stage, i.e. smaller tumours, test for linear trend ($P$ = 0.007). In spite of a lower T-stage among HRT users, no difference concerning lymph node positivity at the time of diagnosis was found between ever-users and never-users (Table 4).

ER status was successfully analysed in 730 breast tumours; 244 women had ER-negative tumours and 486 women had ER-positive tumours. In a Cox regression model it was shown that ER positivity was associated with less risk of dying (RR 0.76; 95% CI 0.68–0.85; $P$ < 0.0001) and so was HRT use (RR 0.75; 95% CI 0.62–0.92; $P$ = 0.005), after adjustment for year of diagnosis.

*© 1999 Cancer Research Campaign*

PR status was successfully analysed in 659 breast tumours; 299 women had PR-negative tumours and 360 women had PR-positive tumours. PR positivity was associated with less risk of dying (RR 0.81; 95% CI 0.72–0.91; $P$ = 0.0004) and so was HRT use (RR 0.71; 95% CI 0.57–0.89; $P$ = 0.003), after adjustment for year of diagnosis. When analysing ER-positive and ER-negative tumours separately among women aged 45 years and older at diagnosis, HRT use was borderline significantly associated with longer overall survival in women with ER-positive tumours (log-rank $P$ = 0.050) and also non-significantly associated with longer overall survival (log-rank $P$ = 0.061) in women with ER-negative tumours (Figures 3 and 4). Among women with PR-positive breast tumours, HRT use was significantly associated with longer survival (log-rank $P$ = 0.037), and also in PR-negative breast cancer patients, HRT use was significantly associated with longer survival (log-rank $P$ = 0.035) (Figure 5 and 6).

Comparing overall survival between ever-users and never-users in a regression model and also taking screening versus symptomatic detection into account, 705 women were eligible for analysis, both HRT use and detection by screening were independently significantly associated with longer overall survival ($P$ = 0.039 and $P$ = 0.002 respectively).

### DISCUSSION

The main finding in this study was that the overall survival among breast cancer patients was significantly longer in women who had ever been treated with HRT before diagnosis compared with women who never used HRT. This was true both for women with ER-positive and ER-negative as well as PR-positive and PR-negative tumours. Women whose tumours were detected by screening also had significantly longer overall survival compared with women whose cancers were symptomatic at diagnosis. When adjusted for mode of detection, T-, N- and M-stage, age at diagnosis and tumour receptor status, ever-use of HRT was still significantly associated with longer overall survival after breast cancer diagnosis. No separate analyses were done on specific causes of deaths.

Others have found that prior post-menopausal oestrogen replacement therapy does not compromise breast cancer survival (Bergkvist et al, 1989; Strickland et al, 1992). In the Nurses Health Study it was shown that overall mortality among women using HRT was lower than among non-users, but that the survival benefit diminished with longer duration of use mainly due to increased risk of dying from breast cancer, and was lower for women with a low risk of coronary disease (Grodstein et al, 1997). Among women using HRT for more than 10 years, the risk of dying of breast cancer was increased by 43%. In addition, they found no apparent overall survival benefit from past hormone use, which is in contrast with our finding of a longer overall survival among women who had ever used HRT prior to their breast cancer diagnosis irrespective of duration. We did not find any significant differences in overall survival between women using HRT for 5 years or more versus a shorter period of use.

We found that HRT users were more likely than never-users to have their tumours detected at a lower T-stage, whereas there was no difference in lymph node spread. In contrast with our finding, Jones et al (1994) found that the percentage of all HRT users with involved lymph nodes (23%) were significantly lower than the percentage of non-users (44%) among 258 non-users and 39 HRT users in Australia. Another study from a Swedish population reported that HRT users had non-significantly smaller tumours and also a non-significantly lower risk of lymph node involvement (Magnusson et al, 1996). Although there were no significant differences between HRT users and never-users in the present study when comparing mode of diagnosis, a significantly larger proportion of never-users had missing information on mode of diagnosis. We have no clear reason for this discrepancy, but one hypothesis could be that HRT users may provide more accurate information about how their tumours were detected. However, we have no reason to believe that the true mode of detection in women with missing information should be distributed unevenly between HRT users and never-users. It is possible that HRT users are more concerned about their health, and that this attitude would be reflected in more careful overall health surveillance leading to better survival. This matter has been previously discussed by other groups (reviewed by Schairer et al, 1994). Further insight into this matter would require a differently designed study.

In our study, women with breast cancer diagnosed at ages 45–60 years, where breast cancer is the main cause of death, we found the survival benefit from HRT use to be 22.5%. This approaches the one seen with mammography screening where a 29% death reduction has been seen among women aged 50–69 years (Nyström et al, 1993).

We consider it an advantage that we interviewed women regarding their hormonal use instead of relying on prescriptions, since there is no guarantee that a woman actually uses the HRT during the intended period, or indeed at all. One study examining the consistency between oral contraceptive use reported by patients and the information in medical records, found that patient information on total duration, numbers of episodes of use, and time since first and last use agreed reasonably well with medical records, while oral contraceptive brand names and duration of use of a specific brand showed less consistency between medical record and patient information (Nischan et al, 1992). Persson et al (1997) found several differences among women who had been prescribed HRT in the choice of complying with the prescription. They found that women denying intake or using HRT short-term (1–72 months) had higher parity, earlier age at first birth and a lower prevalence of hysterectomy or oophorectomy than those complying or exposed long-term. A high level of education was associated with compliance and long-term use, heavy physical exercise and high fibre intake were associated with compliance. In this study we have not distinguished between specific brand names and have asked the women themselves of duration of their HRT instead of using prescription records.

One question raised has been whether HRT users are healthier than non-users prior to use (Yuen et al, 1993; Matthews et al, 1996). One study on HRT compliance (Persson et al, 1997) reported that there were several connections between known risk factors for breast cancer and the use of HRT. We found no significant correlation between height or weight and HRT use. However, it is likely that women who suffer most from oestrogen deficiency also seek help for their symptoms. Lean women are at less risk for developing post-menopausal breast cancer than heavier women (Hunter and Willet, 1993), and they are also more likely to be oestrogen-deficient than heavier women as oestrogen is formed in fatty tissue. We found that leaner and taller women had significantly longer overall survival than shorter and heavier women in the age-group of 45–60 years at breast cancer diagnosis, after adjustment for HRT, T-, N-, M-stage and age at diagnosis. This effect from height and weight was not significant when the whole

© 1999 Cancer Research Campaign 

sample group was studied. In a prospective study it was reported that women with distal forearm fractures had a better survival than expected, mainly due to lower rates of death from malignant tumours and circulatory disease including myocardial infarction (Olsson and Hägglund, 1992). As bone fractures could be a sign of osteoporosis and thus low oestrogen levels, this group of women may also constitute a target group for HRT prescriptions. Forearm fractures may also reflect a physically active life, and the latter study (Olsson and Hägglund, 1992) showed that at least this group of women already had a better survival rate than expected, including less risk of circulatory disease. Therefore one should be cautious when interpreting the effect with an overall longer survival among HRT users. Is the benefit caused by HRT itself or would the group of women prescribed HRT have survival longer even without it?

We found a longer overall survival after breast cancer diagnosis among ever-users of HRT regardless of their tumour receptor status. However, there may be subgroups of women who do not benefit from HRT use which we have been unable to detect in our study. Some of the ever-users may never have developed their breast cancer if they had not used HRT. For a total evaluation of the risk and benefits of HRT a cohort must be followed for a life-time.

In conclusion, we have found that ever-use of HRT before breast cancer diagnosis is significantly associated with longer survival after diagnosis independent of tumour stage, ER and PR status and mode of diagnosis.

### ACKNOWLEDGEMENTS

This study was supported by grants from The Swedish Cancer Society, The Ingvar Kamprad Foundation, The King Gustaf V Jubilee Fund, The Hospital Fund at Lund University Hospital, and Tage Blüchers Foundation.

### REFERENCES

Bergkvist L, Adami H-O, Persson I, Bergström R and Krusemo UB (1989) Prognosis after breast cancer diagnosis exposed to estrogen and estrogen–progestogen replacement therapy. *Am J Epidemiol* **130**: 221–228

Bonnier P, Romain S, Giacalone PL, Laffargue F, Martin PM and Piana L (1995) Clinical and biologic prognostic factors in breast cancer diagnosed during postmenopausal hormone replacement therapy. *Obstet Gynecol* **85**: 11–17

Brinton LA, Hoover R and Fraumeni JF Jr (1986) Menopausal oestrogens and breast cancer risk: an expanded case-control study. *Br J Cancer* **54**: 825–832

Collaborative Group on Hormonal Factors in Breast Cancer (1997) Breast cancer and hormone replacement therapy: collaborative reanalysis of data from 51 epidemiological studies of 52 705 women with breast cancer and 108 411 women without breast cancer. *Lancet* **350**: 1047–1059

Fernö M, Borg Å and Norgren A (1983) A comparison of two steroid receptor assays in breast cancer: dextran coated charcoal and isoelectric focusing. *Anticancer Res* **3**: 243–246

Fernö M, Borg Å and Sellberg G (1986) Enzyme immuno assay of the estrogen receptor in breast cancer biopsy samples. A comparison with isoelectric focusing. *Acta Radiol Oncol* **25**: 171–175

Fernö M, Borg Å and Johansson U (1989) Enzyme immuno assay of progesterone receptor in breast cancer biopsy samples. A comparison with the dextran coated charcoal method. *Acta Oncol* **28**: 19–22

Grodstein F, Stampfer MJ, Colditz GA, Willett WC, Manson JE, Joffe M, Rosner B, Fuchs C, Hankinson SE, Hunter DJ, Hennekens CH and Speizer FE (1997) Postmenopausal hormone therapy and mortality. *N Engl J Med* **336**: 1769–1775

Harding C, Knox WF, Faragher EB, Baildam A and Bundred NJ (1996) Hormone replacement therapy and tumour grade in breast cancer: prospective study in screening unit. *Br Med J* **312**: 1646–1647

Henderson BE, Paganini-Hill A and Ross RK (1991) Decreased mortality in users of estrogen replacement therapy. *Arch Intern Med* **151**: 75–78

Hulley S, Grady D, Bush T, Furberg C, Herrington D, Riggs B and Vittinghoff E (1998) Randomized trial of estrogen plus progestin for secondary prevention of coronary heart disease in postmenopausal women. *JAMA* **280**: 605–613

Hunt K, Vessey M and McPherson K (1990) Mortality in a cohort of long-term users of hormone replacement therapy: an updated analysis. *Br J Obstet Gynecol* **97**: 1080–1086

Hunter D and Willet W (1993) Diet, body size, and breast cancer. *Epidemiol Rev* **15**: 110–132

Jones C, Ingram D, Mattes E and Hahnel R (1994) The effect of hormone replacement therapy on prognostic indices in women with breast cancer. *Med J Aust* **161**: 106–110

Magnusson C, Holmberg L, Norden T, Lindgren A and Persson I (1996) Prognostic characteristics in breast cancers after hormone replacement therapy. *Breast Cancer Res Treat* **38**: 325–334

Matthews KA, Kuller LH, Wing RR, Meilahn EN and Plantinga P (1996) Prior to use of estrogen replacement therapy, are users healthier than non-users? *Am J Epidemiol* **143**: 971–978

Nischan P, Thomas DB and Ebeling K (1992) Accuracy of recall of use of an intrauterine device. *Contraception* **45**: 363–368

Nyström L, Rutqvist LE, Wall S, Lindgren A, Lindqvist M, Ryden S, Andersson I, Bjurstam N, Fagerberg G, Frisell J, Tabor L and Larsson LG (1993) Breast cancer screening with mammography: overview of Swedish randomised trials. *Lancet* **341**: 973–978

Olsson H and Hägglund G (1992) Reduced cancer morbidity and mortality in a prospective cohort of women with distal forearm fractures. *Am J Epidemiol* **136**: 422–427

Persson I, Bergkvist L, Lindgren C and Yuen J (1997) Hormone replacement therapy and major risk factors for reproductive cancers, osteoporosis, and cardiovascular diseases: evidence of confounding by exposure characteristics. *J Clin Epidemiol* **50**: 611–618

Salmon R, Remvikos Y, Ansquer Y and Asselain B (1995) HRT and breast cancer. *Lancet* **346**: 1702–1703

Schairer C, Byrne C, Keyl PM, Brinton LA, Sturgeon SR and Hoover RN (1994) Menopausal estrogen and estrogen-progestin therapy and risk of breast cancer (United States). *Cancer Causes Control* **5**: 491–500

Sigurdsson H, Baldetorp B, Borg Å, Dahlberg M, Fernö M, Killander D and Olsson H (1990) Indicators of prognosis in node-negative breast cancer. *N Engl J Med* **322**: 1045–1053

Squitieri R, Tartter PI, Ahmed S, Brower ST and Theise ND (1994) Carcinoma of the breast in postmenopausal hormone user and nonuser control groups. *J Am Coll Surg* **178**: 167–170

Strickland DM, Gambrell RD Jr, Butzin CA and Strickland K (1992) The relationship between breast cancer survival and prior postmenopausal estrogen use. *Obstet Gynaecol* **80**: 400–404

Sullivan JM, Vander Zwaag R, Hughes JP, Maddock V, Kroetz FW, Ramanathan KB and Mirvis DM (1990) Estrogen replacement and coronary artery disease. Effect on survival in postmenopausal women. *Arch Intern Med* **150**: 2557–2562

Willis DB, Calle EE, Miracle-McMahill HL and Heath CW Jr (1996) Estrogen replacement therapy and risk of fatal breast cancer in a prospective cohort of postmenopausal women in the United States. *Cancer Causes Control* **7**: 449–457

Yuen J, Persson I, Bergqvist L, Hoover R, Schairer C and Adami H-O (1993) Hormone replacement therapy and breast cancer mortality in Swedish women: results after adjustment for 'healthy drug-user' effect. *Cancer Causes Control* **4**: 369–374

© 1999 Cancer Research Campaign
